# A Web-Based Self-Management Support Prototype for Adults With Chronic Kidney Disease (My Kidneys My Health): Co-Design and Usability Testing

**DOI:** 10.2196/22220

**Published:** 2021-02-09

**Authors:** Maoliosa Donald, Heather Beanlands, Sharon E Straus, Michelle Smekal, Sarah Gil, Meghan J Elliott, Gwen Herrington, Lori Harwood, Blair Waldvogel, Maria Delgado, Dwight Sparkes, Allison Tong, Allan Grill, Marta Novak, Matthew Thomas James, K Scott Brimble, Susan Samuel, Karen Tu, Janine Farragher, Brenda R Hemmelgarn

**Affiliations:** 1 Department of Community Health Sciences University of Calgary Calgary, AB Canada; 2 Daphne Cockwell School of Nursing Ryerson University Toronto, ON Canada; 3 Department of Family & Community Medicine University of Toronto Toronto, ON Canada; 4 Can-SOLVE CKD Network Vancouver, BC Canada; 5 London Health Sciences Centre London, ON Canada; 6 Sydney School of Public Health The University of Sydney Sydney Australia; 7 Department of Medicine McMaster University Hamilton, ON Canada; 8 Faculty of Medicine and Dentistry University of Alberta Edmonton, AB Canada

**Keywords:** chronic kidney disease, knowledge-to-action framework, integrated knowledge translation, patient engagement, patient-oriented research, self-management, web-based intervention

## Abstract

**Background:**

Supporting patients to self-manage their chronic kidney disease (CKD) has been identified as a research priority by patients with CKD and those who care for them. Self-management has been shown to slow CKD progression and improve the quality of life of individuals living with the disease. Previous work has identified a need for a person-centered, theory-informed, web-based tool for CKD self-management that can be individualized to a patient’s unique situation, priorities, and preferences. We addressed this gap using an integrated knowledge translation method and patient engagement principles.

**Objective:**

The aim of this study is to conduct systematic co-design and usability testing of a web-based self-management prototype for adults with CKD (nondialysis and nontransplant) and their caregivers to enhance self-management support.

**Methods:**

A multistep, iterative system development cycle was used to co-design and test the *My Kidneys My Health* prototype. The 3-step process included creating website features and content using 2 sequential focus groups with patients with CKD and caregivers, heuristic testing using the 10 heuristic principles by Nielsen, and usability testing through in-person 60-minute interviews with patients with CKD and their caregivers. Patients with CKD, caregivers, clinicians, researchers, software developers, graphic designers, and policy makers were involved in all steps of this study.

**Results:**

In step 1, 18 participants (14 patients and 4 caregivers) attended one of the 2 sequential focus groups. The participants provided specific suggestions for simplifying navigation as well as suggestions to incorporate video, text, audio, interactive components, and visuals to convey information. A total of 5 reviewers completed the heuristic analysis (step 2), identifying items mainly related to navigation and functionality. Furthermore, 5 participants completed usability testing (step 3) and provided feedback on video production, navigation, features and functionality, and branding. Participants reported visiting the website repeatedly for the following features: personalized food tool, my health care provider question list, symptom guidance based on CKD severity, and medication advice. Usability was high, with a mean system usability score of 90 out of 100.

**Conclusions:**

The *My Kidneys My Health* prototype is a systematically developed, multifaceted, web-based CKD self-management support tool guided by the theory and preferences of patients with CKD and their caregivers. The website is user friendly and provides features that improve user experience by tailoring the content and resources to their needs. A feasibility study will provide insights into the acceptability of and engagement with the prototype and identify preliminary patient-reported outcomes (eg, self-efficacy) as well as potential factors related to implementation. This work is relevant given the shift to virtual care during the current pandemic times and provides patients with support when in-person care is restricted.

## Introduction

### Background

Chronic kidney disease (CKD) affects approximately 9% of Canadians [1], with 90% to 95% of the individuals being cared for in the community by primary care [2]. CKD self-management support can slow the progression of the disease and improve the quality of life of those with CKD [3]. International studies of CKD research priority setting involving patients and those who care for them has identified the need for strategies to help patients self-manage their CKD as a top-10 research priority [4-6].

Many individuals with CKD receive self-management education and support through face-to-face interactions in the clinic setting [7]. However, with increasing access to the internet [8] and the nature of pandemics, the potential for an eHealth platform to provide easy access and timely, tailored information and support could be a sustainable way to enhance self-management strategies. eHealth applications can be designed to improve knowledge and self-management behaviors and actively involve individuals in their care [9]. Numerous websites are readily available to support CKD self-management, providing educational programs and support [10-12]. However, patients are infrequently involved in their development, and the websites lack features that combine health information with decision support and/or assist with behavior change [13,14].

Individuals with CKD (nondialysis and nontransplant) have multiple needs that can differ based on the complexity of their illness, health-related knowledge, and confidence in managing the disease. An eHealth CKD self-management support intervention that can be individualized to a patient’s unique situation, priorities, and preferences holds promise for improving their health outcomes and enhancing their quality of life.

### Previous Work

This study is informed by our previous work using a collaborative and systematic approach to determine the best practices for CKD adult self-management support interventions [7,13,15-17]. Specifically, our work is guided by the integrated knowledge translation (IKT) method [18] and the Canadian Institutes of Health Research (CIHR) Strategy for Patient-Oriented Research (SPOR) patient engagement principles [19]. Our knowledge users from the CKD community include patients, caregivers, clinicians, researchers, and policy makers from across Canada. Throughout this work, 4 to 6 patients and caregivers (patient partners) were active members of our research team. The patient partners represent diversity in age, gender, ethnicity, and attitudes toward technology.

### Objectives

The aim of this study is to conduct systematic co-design and usability testing of a web-based, self-management prototype for adults with CKD (nondialysis and nontransplant) and their caregivers to enhance self-management support.

## Methods

### Applying IKT and Patient Engagement to the Broader Program of Work

**Figure 1:**
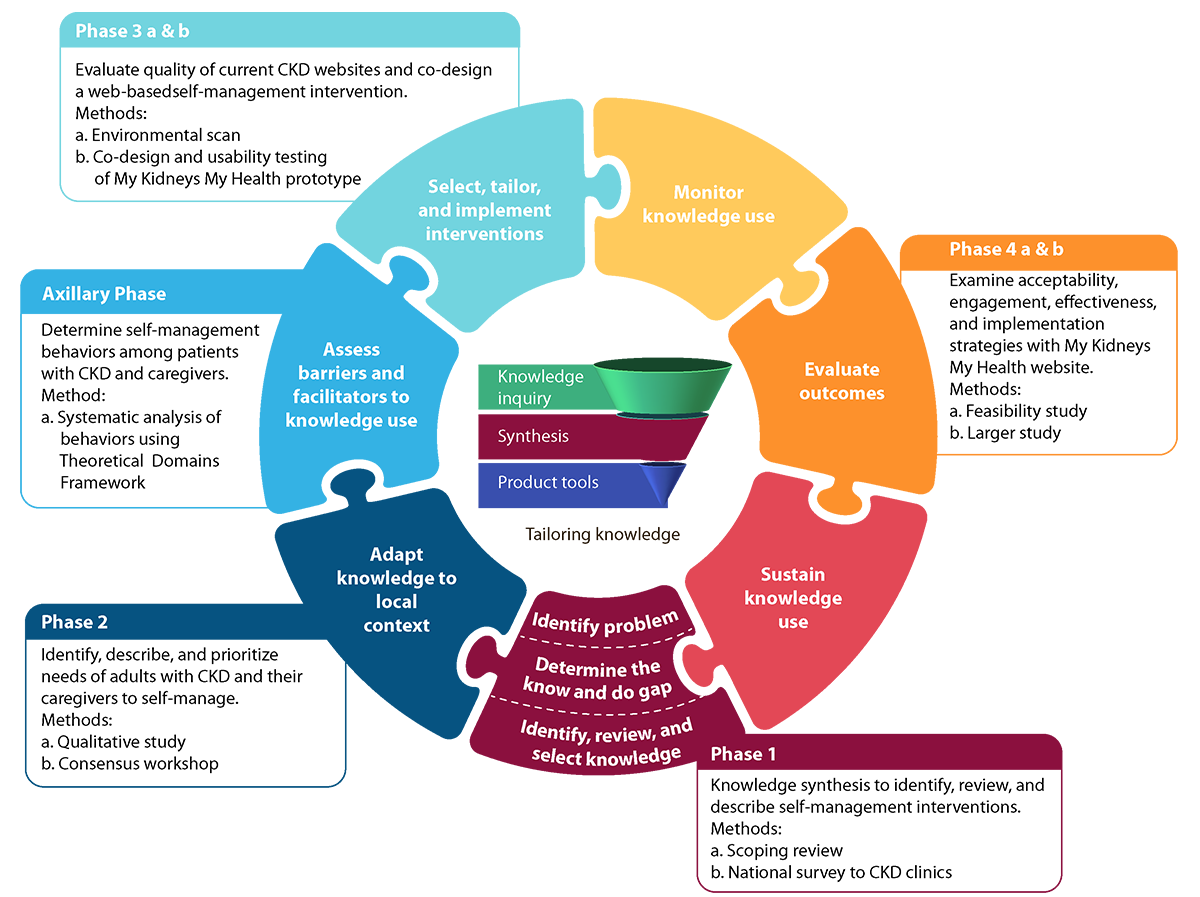
Knowledge-to-action framework and chronic kidney disease self-management project multiphase activities. CKD: chronic kidney disease.

The knowledge-to-action (KTA) framework guided multiphase activities for determining the self-management support intervention for patients with CKD [20]. The KTA framework consists of 2 components: the *knowledge creation* funnel and the *action cycle*. We mapped our activities to the KTA framework (Figure 1). In phase 1, our patient partners contributed to the conceptualization of the research problem and the identification of relevant literature (including gray literature and patient education resources) for our scoping review [13] and national survey for CKD clinics [7]. Patient engagement with our patient partners and linkages with patients and caregivers in the community helped us identify knowledge gaps within the current literature and care to support CKD self-management. In phase 2, through 6 focus groups and 11 interviews (33 patients and 15 caregivers), we identified the perceived self-management needs (eg, empowerment through knowledge and tangible supports), including the potential for an eHealth tool (ie, activation through information sharing by the way of accessible, relevant, timely, and appropriate amount of advice) [16]. Our sample included participants with a diverse range of demographic and clinical characteristics, recruited through purposive sampling. We prioritized these needs during a consensus workshop with key stakeholders and identified the features and content for a web-based eHealth tool [15]. The stakeholders included 24 participants from across Canada: 11 patients, 6 caregivers, 2 nurses, 1 dietitian, 1 pharmacist, 1 policy maker, 1 primary care physician, and 1 nephrologist. The preferred features included visuals, the ability to enter and track health information and interact with health care providers, *on-the-go* access, links to resources, and access to personal health information. An axillary study was conducted to assess patient and caregiver barriers and facilitators for the self-management of CKD [17]. In phase 3a, an environmental scan of CKD self-management websites [14] identified a gap between web-based applications and our population’s self-management support needs, necessitating the co-design of a website. This study (phase 3b) involved the co-design (involving patient partners, patient and caregiver study participants, clinicians, researchers, software developers, graphic designers, and policy makers) and usability testing of the *My Kidneys My Health* web-based prototype that is compatible with mobile devices to support self-management.

The *My Kidneys My Health* prototype was co-designed and tested using a 3-step system development cycle [21] and agile methodologies [22]. These approaches emphasize collaboration with our system users (patients and caregivers) and the flexibility to respond to the changes and needs of users throughout the process. The 3 steps included the creation of website features and content, heuristic testing, and usability testing. Ethics approval was obtained from the University of Calgary Conjoint Health Research Ethics Board (REB19-0002). All participants provided written consent before their participation.

### Step 1: Creation of Website Features and Content

The purpose of step 1 was to evaluate the website features identified during the consensus workshop [15] and present the website components that may deliver the 4 behavior-change strategies we identified in our axillary study (ie, education, modeling, persuasion, and environmental restructuring) [17]. In creating the website features and content, we considered mobile device compatibility. In this step, we built relevant content based on the 8 topic areas identified by patients and caregivers in our previous work (ie, understanding CKD, diet, symptoms, medication and alternate treatments, finances, mental and physical health, travel, and work and school considerations) [16]. We included relevant, credible, and nonproprietary content identified in our environmental scan of CKD websites [14]. Content was also acquired from the Kidney Disease: Improving Global Outcomes CKD guidelines [23] and other relevant agencies (eg, Diabetes Canada, Canadian Cardiovascular Society), along with content expert input from clinicians specializing in CKD care (ie, dietitians, pharmacists, social workers, nurses, nephrologists, and occupational therapists). To ensure reliable, objective, and valid information, we followed the Health on the Net guidelines [24]. The Web Content Accessibility Guidelines were followed to deliver content accessible for individuals with low vision or other accessibility needs [25]. Efforts were made using the Microsoft Office software to ensure that the readability level met that of the general population (ie, grade 7 or less).

**Figure 2:**
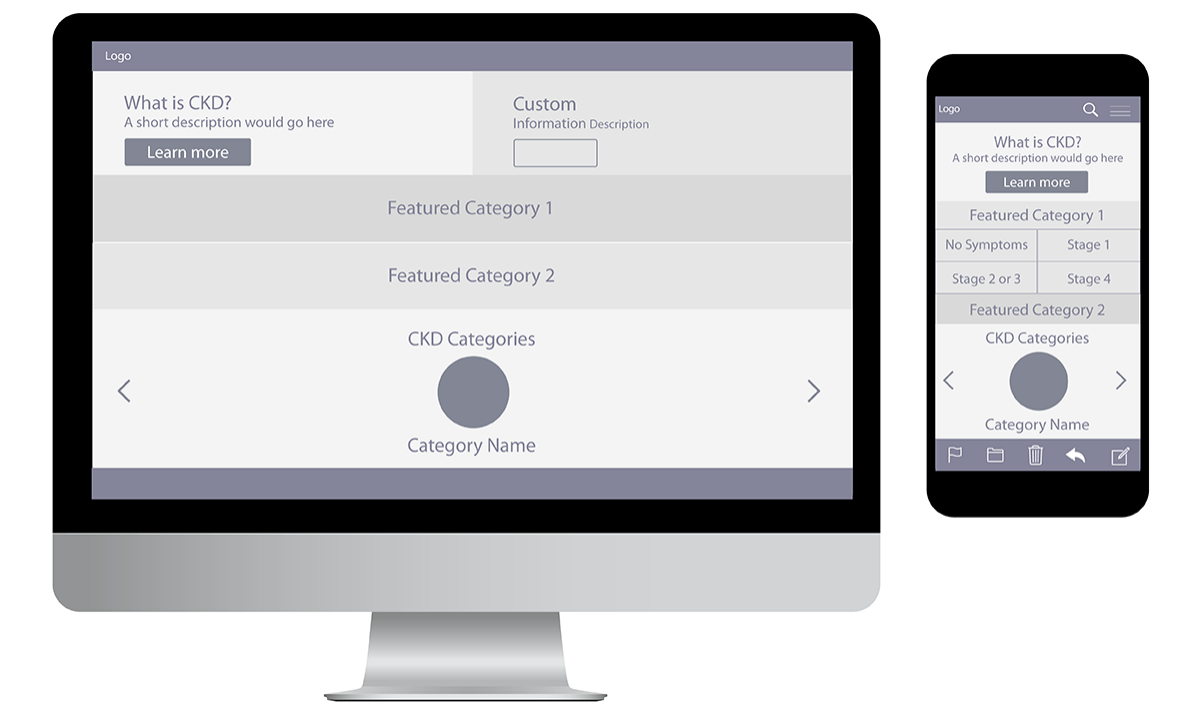
Website wireframes.

**Figure 3:**
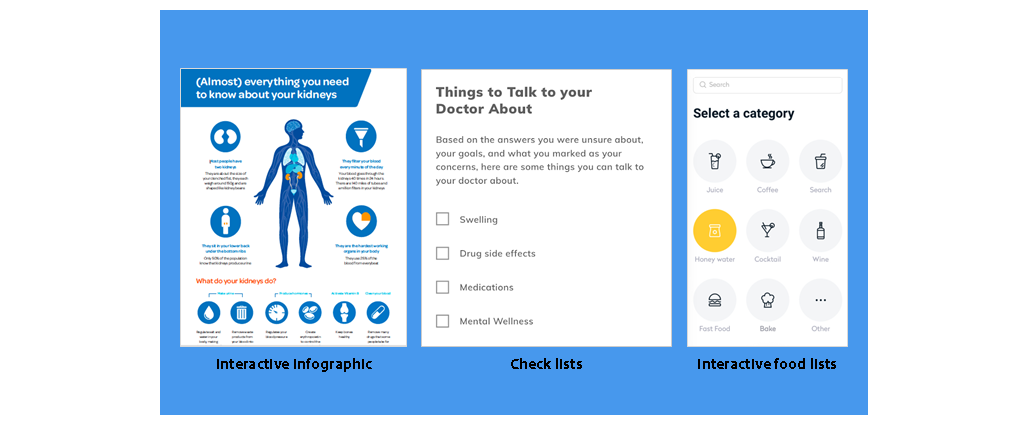
Website visual conceptualizations.

Wireframes and mood boards were developed based on previously constructed personas [15] to aid developers by providing detailed descriptions of user goals, motivations, and behaviors (Figure 2). Research team members reviewed iterations of wireframes and mood boards (ie, color palettes and typography) to inform visual feature conceptualizations (Figure 3).

Using purposive sampling, individuals from CKD and general nephrology clinics in Calgary, Alberta, were invited to participate if they met the following eligibility criteria: were English speaking, were aged 18 years or above, were able to provide informed consent, and were aware of their diagnosis of CKD (stages 1-5, not currently on dialysis, and not a previous kidney transplant recipient) regardless of the cause or duration of CKD. Informal caregivers (eg, family members and friends) of individuals with CKD were also eligible. Participants completed 2 questionnaires: (1) a demographic form and (2) the eHealth Literacy Scale (eHEALS). The eHEALS is an 8-item self-report tool measuring individuals' perceived ability to find, evaluate, and apply eHealth information [26]. The overall scores range from 8 to 40, with a higher score indicating higher self-perceived eHealth literacy [26].

A total of 2 sequential focus groups were facilitated by MD (primary author) to obtain participant feedback and preferences regarding the proposed features. The initial focus group reviewed the visual feature conceptualizations and the prioritized features (ie, voting technique using Monopoly Money to determine the value of the individual features) to be developed in further detail for the initial click-through website prototype. The focus group questions addressed accessibility, adoption, sustainability, and overall strengths/weaknesses of the key features (Multimedia Appendix 1). The second focus group reviewed the initial prototype, and participants were instructed to provide their input on the proposed website pages and functional elements. Questions focused on individual webpage presentation (eg, clarity and missing items) and functional elements (eg, drag and drop, check boxes, and tailored features; Multimedia Appendix 2). Research team members, software developers, and graphic designers developed the semistructured interview guides.

The focus groups were audio recorded and transcribed verbatim by a transcriptionist with experience in qualitative research. A total of 2 research members (MD and MS) completed a descriptive synthesis of the voting results and focus group transcripts. The research team members reviewed the findings from both sessions, and appropriate refinements were made to the prototype.

### Step 2: Heuristic Testing

Heuristic testing based on the 10 heuristic principles by Nielsen [27] was undertaken independently by 5 reviewers (3 research team members [MS, SG, and SA], 1 human factor specialist [JH], and 1 software developer [MP]) to identify system and design issues with the prototype viewed on computer and mobile devices. The areas of focus included navigation, information architecture, search, forms and data entry, trust and credibility, writing and content quality, page layout, and visual design. The reviewers were asked to rate each website page using the following scoring: −1 (does not comply), +1 (complies), or 0 (partially complies). If an item within the focus area was not relevant, the reviewers were instructed to leave the rating blank. Reviewers provided comments to explain their ratings. The reviewers met to discuss the ratings, and consensus was reached through discussion regarding the items to be addressed before usability testing.

### Step 3: Usability Testing

Purposive sampling, using the same recruitment strategy, inclusion criteria, and questionnaires (ie, demographic and eHEALS), was used as previously described in step 1. Potential participants had to have no previous exposure to the development of the tool. Acknowledging that usability testing with 3 to 5 participants can identify 85% of usability issues, we aimed to recruit this number of participants [28].

In-person 60-minute interviews were conducted by MD (primary author) to identify issues with the prototype interface and strategies and paths that participants use, including the time spent in completing tasks. Participants were interviewed in their own natural environment (ie, where they would likely use the *My Kidneys My Health* website), using their preferred devices (ie, desktop, laptop, tablet, and mobile phone).

Participants worked through 5 scenarios (Multimedia Appendix 3) while engaging in a think-aloud protocol (ie, participants communicated their thought processes verbally while performing prespecified tasks) and responded to a series of open-ended questions about the features, format, interface, and content [29]. The System Usability Scale (SUS) was completed by each participant to access the perceived usability [30].

Interviews were audio recorded and transcribed verbatim by a transcriptionist with experience in qualitative research. The interview transcripts were independently analyzed using directed content analysis by 2 research team members (MD and MS) applying a deductive approach to categorize what was useful and the areas to enhance [31]. This list was used by software developers to refine the *My Kidneys My Health* website prototype.

## Results

### Step 1: Creation of Website Features and Content

A total of 18 participants (14 patients and 4 caregivers) attended one of the 2 sequential focus groups. Tables 1 and 2 provide the participant characteristics. Among the participants, 72% (13/18) were male, 67% (12/18) were over the age of 65 years, 61% (11/18) had a secondary or postsecondary education, and 67% (12/18) used the internet for more than 10 hours per week. Overall, 86% (12/14) of the patient participants had at least one comorbidity, 79% (11/14) were diagnosed with CKD in the past 10 years, 72% (10/14) had less severe CKD, and 72% (10/14) perceived their health status as *good*. A total of 16 participants completed the eHEALS, with a mean score of 26.7 (range 15-32), demonstrating a variety of perceived ability to use information technology for health.

**Table 1:** Focus group participant characteristics (N=18).

Patient and caregiver characteristics		Participants, n (%)
**Role**		
	Patient	14 (78)
	Caregiver	4 (22)
**Gender**		
	Male	13 (72)
	Female	5 (28)
**Age (years)**		
	Under 50	2 (11)
	50-64	4 (22)
	65-74	5 (28)
	≥75	7 (39)
**Marital status**		
	Common law	1 (6)
	Married	13 (72)
	Single or widowed	4 (22)
**Geographical location (population)**		
	<500,000 (rural)	5 (28)
	≥500,000 (urban)	13 (72)
**Level of education**		
	Primary (≤grade 12)	7 (39)
	Secondary (college, university, or trade school)	9 (50)
	Postsecondary graduate	2 (11)
**Level of employment**		
	Full-time	4 (22)
	Other (home duties, unemployed, student, or retired)	14 (78)
**Ethnicity**		
	White	18 (100)
**Electronic devices commonly used (can use multiple devices)**		
	Mobile phone	10 (56)
	Tablet	11 (61)
	Laptop	9 (50)
	Desktop	8 (40)
**Electronic devices used (mobile phone, tablet, laptop, and desktop)**		
	1	4 (22)
	2	9 (50)
	3	4 (22)
	4	1(6)
**Internet use (hours per week)**		
	<4	4 (22)
	4-9	2 (11)
	10-15	8 (45)
	>15	4 (22)

**Table 2:** Self-reported patient clinical characteristics (N=14).

Self-reported patient clinical characteristics		Participants, n (%)
**Duration of CKD^a^ diagnosis (years)**		
	≤5	9 (65)
	6-10	2 (14)
	≥11	3 (21)
**Cause of CKD**		
	Diabetes and/or high blood pressure	5 (36)
	Glomerulonephritis (eg, Immunoglobulin A nephropathy, lupus)	4 (29)
	Other (eg, sepsis, hereditary, obstruction)	2 (14)
	Unknown	3 (21)
**Comorbidities (can have multiple comorbidities)**		
	Diabetes	2 (14)
	High blood pressure	11 (79)
	Cardiovascular disease	1 (7)
	None	2 (14)
**Number of comorbidities**		
	0	2 (14)
	1	10 (72)
	2	2 (14)
**Stage of CKD (eGFR^b^, mL/min/1.73 m^2^)**		
	30-60	5 (36)
	15-29	5 (36)
	<15	1 (7)
	Unknown	3 (21)
**Perceived health status**		
	Excellent	0 (0)
	Very good	1 (7)
	Good	10 (72)
	Fair	2 (14)
	Poor	1 (7)

^a^CKD: chronic kidney disease.

^b^eGFR: estimated glomerular filtration rate.

Participants from the first focus group endorsed the following features: interactive infographics about CKD, interactive food list and food label, customizable symptom list based on CKD severity, searchable medication list, and a screening tool for depression. Participants also suggested that the diet information should be customizable using food and nutrient filters to provide an individualized list of food items they could eat. They also recommended a personal list of questions for their health care provider based on their current information needs. The second focus group identified items on page layouts that were confusing or missing and provided suggestions for making them appropriate for users. They also provided visual and navigation suggestions as well as suggestions to incorporate video, text, audio, and visuals to convey information.

**Figure 4:**
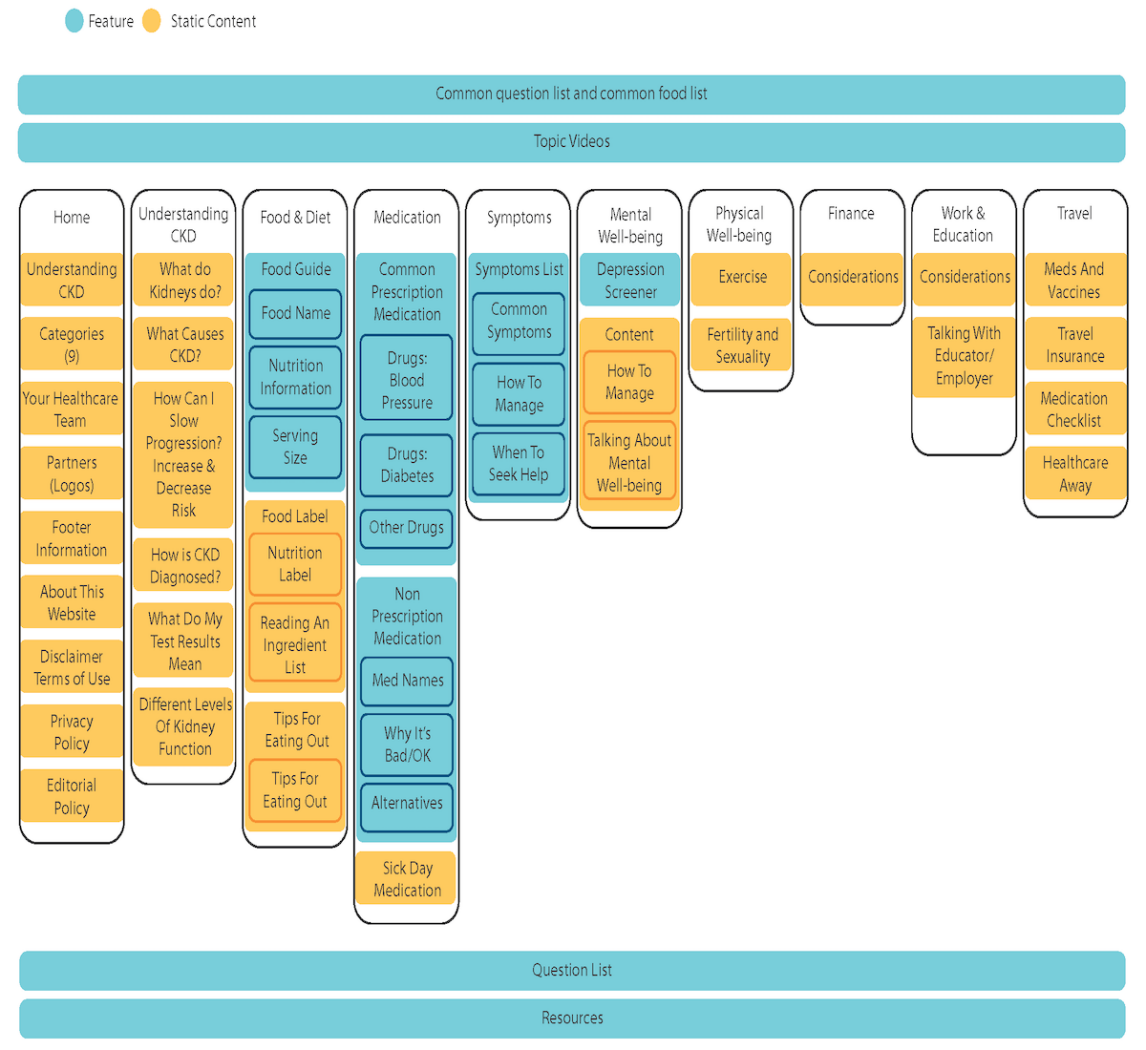
Website sitemap.

Further modifications were made to the *My Kidneys My Health* prototype and are represented on the website sitemap (Figure 4). The prototype consisted of 3 main components: videos, static content, and 4 interactive features. The first interactive feature included a *food guide/tool* that could be tailored to filter foods by nutrient content and a searchable database of over 400 common foods to create a personalized CKD-friendly food list that could be saved or emailed. The second feature was a search function specific to symptoms by CKD severity and medications by categories (ie, prescription, nonprescription, and sick day). The third feature was a validated screening tool for depression (ie, Patient Health Questionnaire-2) [32], and the fourth feature was a personalized list of questions to enhance communication with health care providers. The personalized question list could also be saved or emailed.

### Step 2: Heuristic Testing

Items that scored −1 (does not comply) and 0 (partially complies) were categorized by reviewers into *must* (ie, need to be addressed), *should* (ie, would like to be addressed), and *could* (ie, not necessary at this stage of development), with most items related to navigation and functionality; for example, the inclusion of a website navigation bar to move between website pages and the use of filters to personalize food choices for diet restrictions. Further modifications were made to the prototype before usability testing.

### Step 3: Usability Testing

A total of 5 participant interviews (4 patients and 1 caregiver) were conducted; the participant characteristics are presented in Tables 3 and 4. After completing 5 interviews, we noted similar responses among participants with regard to the design, content, and navigation of the prototype. Among the participants, 60% (3/5) were male and were aged 65 years or above. A total of 80% (4/5) of the participants had postsecondary education and spent more than 15 hours a week on the internet. Furthermore, 75% (3/4) of the patients had less severe CKD. The mean total score for eHEALS was 29.6 (range 23-40).

**Table 3:** Usability testing participant characteristics (N=5).

Characteristic		Participants, n (%)
**Role**		
	Patient	4 (80)
	Caregiver	1 (20)
**Gender**		
	Male	3 (60)
	Female	2 (40)
**Age (years)**		
	Under 50	2 (40)
	50-64	0 (0)
	65-74	3 (60)
	≥75	0 (0)
**Marital status**		
	Common law	1 (20)
	Divorced	1 (20)
	Married	1 (20)
	Single	2 (40)
**Geographical location (population)**		
	<500,000 (rural)	1 (20)
	≥500,000 (urban)	4 (80)
**Level of education**		
	Primary (≤grade 12)	1 (20)
	Postsecondary (college, university, and trade school)	4 (80)
	Graduate	0 (0)
**Level of employment**		
	Full-time	1 (20)
	Part-time	1 (20)
	Retired	3 (60)
**Ethnicity**		
	White	4 (80)
	Visible minority	1 (20)
**Electronic devices used (mobile phone, tablet, laptop, and desktop)**		
	1	1 (20)
	2	1 (20)
	3	2 (40)
	4	1 (20)
**Internet use (hours per week)**		
	<4	0 (0)
	4-9	1 (20)
	10-15	0 (0)
	>15	4 (80)

**Table 4:** Self-reported patient clinical characteristics (N=4).

Characteristics		Participants, n (%)
**Duration of CKD^a^ diagnosis (years)**		
	≤5	2 (40)
	6-10	1 (20)
	≥11	1 (20)
**Perceived level of health**		
	Excellent	0 (0)
	Very good	2 (40)
	Good	1 (20)
	Fair	1 (20)
	Poor	0 (0)
**Stage of CKD (eGFR^b^, mL/min/1.73 m^2^)**		
	30-60	3 (75)
	15-29	1 (25)
	<15	0 (0)
**Cause of CKD**		
	Diabetes	1 (20)
	Diabetes and hypertension	1 (20)
	Hypertension	1 (20)
	Alport syndrome	1 (20)

^a^CKD: chronic kidney disease.

^b^eGFR: estimated glomerular filtration rate.

The usability categories reported on included video production, navigation, features and functionality, and branding. Participants reported that the following features were important for them to return to the website: personalized food tool, my health care provider question list, symptom guidance based on CKD severity, and medication advice. Usability was high, with a mean SUS score of 90 out of 100. Participants identified future considerations, including linking the website with a patient portal to upload personal health information and addressing other medical conditions such as diabetes (eg, add fat and sugar nutrient values to each of the food items).

## Discussion

### Principal Findings

Using an iterative 3-step system development cycle, we engaged patients and caregivers in the co-design and usability testing of the *My Kidneys My Health* website prototype. The application of the KTA framework enabled us to co-create a patient-facing CKD, self-management intervention grounded in evidence, patient preferences, and theoretical frameworks.

On the basis of patient and caregiver preferences, our website has the ability to inform through text, visuals, audio, and video (eg, CKD-related information); guide through interactive tools to make personalized recommendations to manage CKD and enhance quality of life (eg, food tool, screen for depression); and engage in communication with health care providers (eg, personalized list of questions for health care providers that can be emailed or saved) and peers (eg, links to nationwide support).

CKD self-management support interventions may consist of a variety of components, including education and management of the condition, information about resources, provision of action plans and equipment, monitoring of the condition, training, social support, and lifestyle advice [33]. A recent systematic review by Shen et al [34] evaluated the implementation and effectiveness of eHealth self-management interventions for patients with CKD. They identified 23 studies, with 9 studies using more than 1 eHealth technology (eg, telemedicine and wearable device). Only 3 studies reported using a computerized system (internet-based system where data are entered by a patient or provided by the system) with multiple functionality components (ie, record, communicate, alert, educate, and display) and a variety of intervention components (eg, education, plan/goals, and self-monitoring). The authors reported that overall eHealth self-management interventions were highly feasible, usable, and acceptable for patients with CKD; however, the 3 computer interventions were not developed using theory or in partnership with patients.

Although eHealth CKD self-management support interventions are burgeoning, interventions rarely consider a behavioral theoretical framework to investigate individual behavior change [7,34]. Our previous work explored the self-management behaviors of patients with CKD and their caregivers using the theoretical domains framework [17]. The *My Kidneys My Health* prototype considers some of these behavior-change strategies (ie, education, modeling, persuasion, and environmental restructuring) [17]. Incorporating multiple behavior-change techniques can improve the effectiveness of eHealth interventions [35]. In terms of education, our website includes information on CKD-related information (topics identified by patients and caregivers), in addition to the consequences of certain health behaviors (eg, explaining *why* it is important to follow medication and diet advice). Although the website does not directly address modeling (ie, providing examples of others living with CKD), the website directs users to the Kidney Foundation of Canada (KFOC) website where there are stories and peer support opportunities. The *My Kidneys My Health* prototype provides an element of persuasion by providing credible information and resources. Finally, we address environmental restructuring by providing individuals with a web-based option where information and resources are accessible from any place users choose to engage in self-management support.

Meaningful patient involvement in our co-design process led to an intuitive and functional prototype. Our website architecture was logical to patients and caregivers and allowed users to follow their own personal journey, where they could have different paths depending on their needs. Participants indicated that they wanted information using a variety of formats (text, visuals, audio, and video) to address sensory needs (eg, vision and hearing deficits) and to be culturally sensitive. The system features can be tailored for each individual’s context and changing life circumstances. For example, the interactive food tool can be used to create a personal list of foods based on restrictions at various times in the patient’s health journey. Another unique feature of the *My Kidneys My Health* website is the personalized list of questions for their health care providers. Many patients with CKD, especially in the early stages, have difficulty comprehending the impact the illness can have on their lives. Involving patients in conversations with health care providers can increase their knowledge and develop confidence in managing their CKD [36]. An effective strategy is to use a question prompt sheet (prepared list of questions that the patients can review before their health care visit) [37]. Preliminary testing of question prompt sheets in nephrology has been undertaken [37,38] and shown to be feasible. However, they are limited in their current form, as they include preprescribed questions for selecting topic areas. The *My Kidneys My Health* prototype allows users to select relevant questions, save, and email questions at any time to themselves, family, and/or health care providers. Preloaded questions created by patients and caregivers are available under each topic area, and users can add or delete questions to create their own unique health-related question list. This list can prepare patients to initiate or enhance conversation with a variety of health care providers and empower them to take an active role in their care.

### Strengths and Limitations

This study has several strengths. These include a person-centered, theory-informed, IKT approach where stakeholders were engaged throughout the process to build a multicomponent web-based self-management support for patients with CKD. However, this study also has limitations. The patient participants were from local CKD and general nephrology clinics, and the majority were older (aged >65 years) with less severe CKD. The website appears to be useful and accessible to older adults; however, the participants do not represent younger adults with CKD. Most of our participants were male, had high educational attainment, and indicated that they used the internet frequently (>10 hours a week). Selection bias is possible and may have favored individuals who had a relatively high education levels, were motivated to engage in self-management, and/or had an interest in technology-based interventions to manage their health. We also recognize the limited number of caregivers included, although the primary focus was on patients with CKD. Overall, other populations with CKD may identify different preferences for the website features based on their needs.

### Conclusions

eHealth interventions show promise to support self-management for patients with CKD and to aid in slowing down or preventing the progression of the disease to kidney failure. We successfully co-designed and tested the usability of a multifaceted, self-management, web-based prototype for patients with CKD. It was guided by the preferences of patients with CKD and their caregivers using a systematic iterative process. Our eHealth tool informs, activates, and promotes communication with the intent to empower patients in their health care. Although many individuals may be willing and capable of using a web-based resource such as *My Kidneys My Health*, it is one of many strategies to enhance CKD self-management. The next phase of our work is to complete a feasibility study to determine the acceptability of and engagement with the prototype and identify preliminary patient-reported outcomes (eg, self-efficacy) and potential factors related to implementation. Our work is relevant given the shift to virtual care during the current pandemic and can support patients in managing their CKD.

## Appendix

Multimedia Appendix 1 Interview Guide 1.

Multimedia Appendix 2 Interview Guide 2.

Multimedia Appendix 3 Usability scenarios.

## Abbreviations

CKD: chronic kidney disease
eHEALS: eHealth Literacy Scale
IKT: integrated knowledge translation
KTA: Knowledge-To-Action
