# Supplementary material for: A Web-Based Self-Management Support Prototype for Adults With Chronic Kidney Disease (My Kidneys My Health): Co-Design and Usability Testing
Source: JMIR Form Res. 2021 Feb 9;5(2):e22220. doi: 10.2196/22220 (PMC7902181; doi:10.2196/22220)
Supplement: Multimedia Appendix 1 [file formative_v5i2e22220_app1.docx]

| **Interview questions and prompts** | |
| --- | --- |
| Q.1 | **Feature adoption/acceptability/sustainability**  Think about yourself or others using this feature:   - Do you like anything about this concept? Why? - Is there anything you dislike about this concept?/Why?   - - How could we make it better?   - How would this feature have been useful in the past? Presently? Future? Regarding your needs. |
| Q.2 | **Overall website strengths and weaknesses (Monopoly activity)**  Description of monopoly activity (i.e. each of you will be given $100 in $5 denominations. You can spend it all on one feature, or spend $ among numerous features).  Present features most liked:   - Tell me why you valued ________ feature.   Present features least liked:   - Tell me why you did not value _________feature. - What would make this feature better? Why? |
| Q.3 | **General concepts**  Tell me what you/others do not/may not like about this website? Explain?  Are there any health apps/websites you have used to help manage/learn about CKD in the past month?   - Tell me about them. - What did you like/dislike about them? |
