# Supplementary material for: A Web-Based Self-Management Support Prototype for Adults With Chronic Kidney Disease (My Kidneys My Health): Co-Design and Usability Testing
Source: JMIR Form Res. 2021 Feb 9;5(2):e22220. doi: 10.2196/22220 (PMC7902181; doi:10.2196/22220)
Supplement: Multimedia Appendix 2 [file formative_v5i2e22220_app2.docx]

| **Interview questions and prompts** | |
| --- | --- |
| Q.1 | **Individual website pages**   - Is there anything on this screen that is confusing? Explain. - Is there anything you wish was or wasn’t on this screen? Explain. - What would make this the perfect version of this screen? Explain. |
| Q.2 | **Functional elements**  Which method (e.g. drag and drop, check boxes) does everyone feel would be easiest to use? Why?   - What would make this feature better? Why? - Is there other features that may be more useful? Why? |
| Q.3 | **Tailoring features**  Submenu:   - Would you use this feature? Why/why not? - Is there anything confusing about this feature? Why? - Is there anything you wish was or wasn’t included in this feature? - What would make this perfect? Why?   Personalized lists (topics, questions):   - Would you use this feature? Why/why not? - Is there anything confusing about this feature? Why? - Is there anything you wish was or wasn’t included in this feature? - What would make this perfect? Why? |
