# Supplementary material for: A Web-Based Self-Management Support Prototype for Adults With Chronic Kidney Disease (My Kidneys My Health): Co-Design and Usability Testing
Source: JMIR Form Res. 2021 Feb 9;5(2):e22220. doi: 10.2196/22220 (PMC7902181; doi:10.2196/22220)
Supplement: Multimedia Appendix 3 [file formative_v5i2e22220_app3.doc]

# Usability scenarios

1. Initial diagnosis of chronic kidney disease (CKD)

You (or your family member) has been recently diagnosed with CKD. You want to learn more about what CKD is, specifically how to manage it and slow progression.

1. Depression screen – My Question List

You are concerned about your mental well-being (or your family member’s mental well-being). You want to know what you should do.

1. Food and diet tool – My Food List

You have been told that you (or your family member) needs to modify your (their) diet to be “kidney friendly”, specifically choose foods low in sodium. You want to find out what you (they) can eat (for example, meats and cheese items).

1. Symptoms

Your (your family member’s) kidneys are functioning at 40% (eGFR 40) and you (they) have started to develop symptoms, such as being tired. You are interested in knowing what potential symptoms you (they) may develop and how to manage them.

1. Medications

You (your family member’s) has a caught a cold and has been coughing a few days. You (they) want to take some medications for the cough, but unsure of what you (they) can take.
